# Supplementary material for: Immunization of Children under 2 Years Old in the Coastal Hadhramaut Governorate, Yemen, during Public Health Emergencies: A Trend Analysis of 2013–2020
Source: Vaccines (Basel). 2024 Mar 15;12(3):311. doi: 10.3390/vaccines12030311 (PMC10976025; doi:10.3390/vaccines12030311)
Supplement: Supplementary file 1 [file vaccines-12-00311-s001.zip › vaccines-2789280-supplementary.pdf]

Immunization of Children under 2 Years Old in the Coastal Hadhramaut Governorate, Yemen, during Public Health Emergencies: A Trend Analysis of 2013–2020

Suha Ali Batarfi*, Rosnah Sutan*, Halim Ismail and Abdulla Salem Bin-Ghouth

Supplementary Tables:

Table S1: BCG vaccine coverage in Coastal Hadhramout (2013-2020)

BCG								
**Districts**	**2013**	**2014**	**2015**	**2016**	**2017**	**2018**	**2019**	**2020**
**Al-Mukalla**	99.51	98.46	87.67	100.00	100.00	100.00	100.00	97.59
**Al-Mukalla rural**	59.62	70.99	64.66	74.39	59.38	56.63	44.95	37.10
**Al-Shahr**	82.23	79.31	67.30	79.11	85.97	91.49	96.70	84.37
**Ghail Bawazeer**	89.67	87.21	64.24	86.43	78.88	92.46	100.00	88.08
**Al-Dees**	76.79	76.04	76.72	58.93	99.40	72.67	84.42	83.09
**Al-Ridh and Gosaiar**	78.01	81.83	65.12	84.38	100.00	100.00	98.81	84.79
**Ghail binYumin**	75.00	86.78	76.57	82.06	96.84	95.88	98.44	100.00
**Doan**	76.44	71.58	73.80	68.05	63.75	70.98	67.08	66.78
**Al-Dulaia**	60.00	51.36	34.65	58.92	70.47	73.68	78.23	46.84
**Hajer**	69.31	66.67	47.18	51.45	65.52	86.66	67.60	71.65
**Broome- Mayfa'a**	69.59	60.41	74.03	100.00	100.00	94.62	93.41	76.47
**Yeabeth**	61.02	74.49	59.34	71.10	84.72	68.57	65.32	67.79
**Coastal Hadhramout (Total)**	84.28	83.66	72.59	89.03	93.01	97.37	99.05	84.69

Table S2: IPV vaccine coverage in Coastal Hadhramout (2013-2020)

IPV					
**Districts**	**2016**	**2017**	**2018**	**2019**	**2020**
**Al-Mukalla**	100.00	100.00	100.00	100.00	88.74
**Al-Mukalla rural**	83.33	89.29	72.59	84.56	70.43
**Al-Shahr**	81.82	83.16	100.00	88.24	77.60
**Ghail Bawazeer**	94.97	80.92	83.68	100.00	89.52
**Al-Dees**	42.02	38.81	60.11	54.35	34.66
**Al-Ridh and Gosaiar**	89.76	100.00	100.00	96.11	81.12
**Ghai lbinYumin**	85.39	93.20	96.77	95.70	97.08
**Doan**	44.60	61.13	70.04	70.27	60.46
**Al-Dulaia**	29.39	25.15	34.06	25.40	21.09
**Hajer**	36.35	52.81	83.79	94.17	98.59
**Broome- Mayfa'a**	98.77	100.00	96.15	97.76	79.41
**Yeabeth**	38.23	58.80	59.76	42.12	21.85
**Coastal Hadhramout (Total)**	83.49	85.61	93.16	92.74	78.65

Table S3: MR 1 vaccine coverage in Coastal Hadhramout (2013-2020)

MR1								
**Districts**	**2013**	**2014**	**2015**	**2016**	**2017**	**2018**	**2019**	**2020**
**Al-Mukalla**	82.83	79.92	88.19	82.95	80.04	93.19	100.00	95.81
**Al-Mukalla rural**	89.26	88.46	99.23	93.48	80.95	81.21	86.89	76.75
**Al-Shahr**	73.60	70.61	81.58	64.17	74.12	87.62	88.91	73.47
**Ghail Bawazeer**	81.04	81.92	73.79	58.86	70.49	77.56	91.90	84.50

**Al-Dees**	67.69	61.40	82.97	65.36	65.00	77.56	86.35	74.52
**Al-Ridh and Gosaiar**	72.69	81.61	79.99	77.42	87.58	100.00	82.85	77.33
**Ghail binYumin**	81.82	68.36	79.12	69.61	82.57	91.94	93.59	98.37
**Doan**	84.29	84.89	77.22	78.96	80.89	81.21	78.78	79.49
**Al-Dulaia**	73.18	51.06	59.50	59.06	55.70	74.27	75.67	68.18
**Hajer**	69.05	59.13	50.34	53.24	58.85	56.82	75.42	72.19
**Broome- Mayfa'a**	74.27	65.69	82.58	88.12	100.00	85.38	86.57	79.78
**Yeabeth**	46.77	67.93	69.05	74.36	60.19	99.05	82.66	93.92
**Coastal Hadhramout (Total)**	78.16	75.80	80.50	73.96	77.53	87.15	91.05	84.48

Table S4: MR 2 vaccine coverage in Coastal Hadhramout (2013-2020)

**MR2**								
**Districts**	**2013**	**2014**	**2015**	**2016**	**2017**	**2018**	**2019**	**2020**
**Al-Mukalla**	73.22	68.68	79.38	73.41	66.17	75.35	81.42	77.72
**Al-Mukalla rural**	53.85	63.78	67.90	70.45	73.21	72.07	68.17	58.60
**Al-Shahr**	70.87	62.17	77.42	63.29	62.97	69.76	70.58	65.25
**Ghail Bawazeer**	69.42	61.44	67.48	56.13	54.72	61.49	67.56	73.57
**Al-Dees**	56.28	51.42	46.94	51.31	49.05	66.11	65.34	51.21
**Al-Ridh and Gosaiar**	58.76	67.80	61.05	55.24	61.37	74.63	67.68	62.76
**Ghail binYumin**	56.71	57.02	63.53	71.67	59.96	65.86	76.37	77.15
**Doan**	36.63	37.83	38.97	46.94	45.83	55.91	51.24	59.10
**Al-Dulaia**	22.42	11.21	10.96	10.09	13.89	27.05	20.97	27.65
**Hajer**	18.12	27.65	18.47	28.86	26.88	44.07	34.06	48.81
**Broome- Mayfa'a**	29.97	31.23	35.93	22.53	33.76	41.92	42.91	44.49
**Yeabeth**	22.58	16.41	21.23	50.82	24.54	41.43	33.11	39.19
**Coastal Hadhramout (Total)**	59.35	56.89	62.30	59.03	56.20	65.67	67.23	66.14

Table S5: Rota 1 vaccine coverage in Coastal Hadhramout (2013-2020)

Rota1								
**Districts**	**2013**	**2014**	**2015**	**2016**	**2017**	**2018**	**2019**	**2020**
**Al-Mukalla**	92.74	90.94	94.76	89.58	100.00	100.00	100.00	100.00
**Al-Mukalla rural**	65.54	75.00	85.49	92.88	83.63	76.19	93.85	81.32
**Al-Shahr**	85.25	82.04	83.74	63.12	90.54	96.17	100.00	92.45
**Ghail Bawazeer**	88.97	86.15	79.02	69.50	100.00	94.13	100.00	95.16
**Al-Dees**	70.64	72.02	74.88	61.07	98.81	100.33	100.00	88.77
**Al-Ridh and Gosaiar**	80.59	88.44	78.00	83.74	97.81	100.00	100.00	93.33
**Ghail binYumin**	72.84	84.53	80.59	73.24	100.00	100.00	100.00	100.00
**Doan**	74.52	84.65	87.35	81.65	91.73	88.12	90.78	85.99
**Al-Dulaia**	33.48	40.30	47.22	57.46	94.01	90.94	100.00	95.71
**Hajer**	49.60	52.65	43.36	53.69	64.79	94.57	95.10	96.75
**Broome- Mayfa'a**	60.09	54.84	67.50	81.02	91.67	95.51	96.02	84.68
**Yeabeth**	56.99	72.98	81.07	87.18	85.19	100.00	100.00	100.00
**Coastal Hadhramout (Total)**	80.19	81.93	82.32	77.83	95.85	99.08	108.52	98.92

Table S6: Rota 2 vaccine coverage in Coastal Hadhramout (2013-2020)

Rota2								
**Districts**	**2013**	**2014**	**2015**	**2016**	**2017**	**2018**	**2019**	**2020**
**Al-Mukalla**	90.69	86.19	91.78	88.55	100.00	94.35	100.00	100.00
**Al-Mukalla rural**	66.99	68.91	84.10	85.76	82.44	77.09	95.90	79.57
**Al-Shahr**	82.89	83.04	83.15	61.11	87.24	94.59	97.28	85.09
**GhailBawazeer**	83.10	84.10	74.33	66.72	82.70	86.54	100.00	92.52
**Al-Dees**	67.82	61.53	68.87	53.21	80.71	90.56	94.57	77.17
**Al-Ridh and Gosaiar**	80.16	80.65	76.89	79.58	100.00	100.00	97.26	88.31
**GhailbinYumin**	69.59	72.37	72.75	69.41	97.61	100.00	100.00	100.00
**Doan**	71.82	79.26	81.12	71.10	82.14	87.35	86.58	85.70
**Al-Dulaia**	25.30	28.94	33.33	45.32	89.18	82.02	100.00	93.06

**Hajer**	35.05	41.67	33.11	52.80	57.40	82.91	89.27	86.80
**Broome- Mayfa'a**	51.90	51.32	64.07	68.06	86.44	75.00	90.80	67.52
**Yeabeth**	46.77	57.07	70.33	75.99	97.69	100.00	94.82	100.00
**Coastal Hadhramout (Total)**	76.80	76.62	78.21	73.81	91.33	92.51	100.00	93.64

Table S7: Pentavalent 1 vaccine coverage in Coastal Hadhramout (2013-2020)

**Pentavalent 1**								
**Districts**	**2013**	**2014**	**2015**	**2016**	**2017**	**2018**	**2019**	**2020**
**Al-Mukalla**	97.86	95.02	100.00	94.58	100.00	100.00	100.00	100.00
**Al-Mukalla rural**	87.66	96.47	94.29	100.00	86.16	80.05	96.04	81.85
**Al-Shahr**	86.54	83.26	85.31	75.25	90.86	93.25	99.68	93.34
**Ghail Bawazeer**	94.66	88.91	82.69	76.62	84.96	94.63	100.00	95.07
**Al-Dees**	83.21	83.42	83.58	88.33	100.00	100.00	99.28	88.77
**Al-Ridh and Gosaiar**	91.29	93.28	87.84	85.16	100.00	100.00	100.00	94.55
**Ghail binYumin**	91.34	95.28	80.69	85.29	100.00	100.00	100.00	100.00
**Doan**	94.90	96.52	100.00	99.16	94.82	93.20	95.51	90.01
**Al-Dulaia**	81.97	87.12	93.13	85.53	97.95	100.00	100.00	96.59
**Hajer**	92.72	94.71	73.65	76.40	78.54	100.00	97.40	96.86
**Broome- Mayfa'a**	77.49	75.37	92.85	100.00	100.00	100.00	97.39	87.99
**Yeabeth**	87.37	97.98	94.37	94.87	84.95	91.67	100.00	100.00
**Coastal Hadhramout (Total)**	100.00	91.42	91.66	89.34	97.47	100.00	100.00	100.00

Table S8 : Pentavalent 2 vaccine coverage in Coastal Hadhramout (2013-2020)

Pentavalent 2								
Districts	2013	2014	2015	2016	2017	2018	2019	2020
Al-Mukalla	93.69	91.79	98.00	90.48	100.00	100.00	100.00	100.00
Al-Mukalla rural	87.82	90.87	91.36	93.64	88.10	79.15	98.09	76.21
Al-Shahr	83.92	84.73	85.68	72.05	89.80	90.15	95.58	86.51
Ghail Bawazeer	89.14	88.50	79.40	75.00	84.22	86.98	100.00	92.70
Al-Dees	79.74	76.81	83.09	82.50	94.76	98.56	99.64	77.17
Al-Ridh and Gosaiar	86.83	94.52	85.03	88.10	100.00	100.00	96.95	86.14
GhailbinYumin	89.72	89.02	86.67	83.82	100.00	100.00	100.00	100.00
Doan	92.81	96.40	94.18	91.13	91.49	91.43	90.96	88.42
Al-Dulaia	81.82	86.52	93.27	84.80	91.37	100.00	100.00	91.92
Hajer	76.19	83.47	62.27	70.92	69.69	89.03	98.96	92.75
Broome- Mayfa'a	81.73	79.91	90.82	100.00	100.00	87.05	89.55	74.75
Yeabeth	85.48	91.92	89.26	90.21	77.55	83.81	94.82	100.00
Coastal Hadhramout (Total)	88.51	89.46	89.22	86.05	94.35	99.51	100.00	94.20

Table S9: Pentavalent 3 vaccine coverage in Coastal Hadhramout (2013-2020)

Pentavalent 3								
**Districts**	**2013**	**2014**	**2015**	**2016**	**2017**	**2018**	**2019**	**2020**
**Al-Mukalla**	91.45	90.03	94.21	93.00	99.02	100.00	100.00	100.00
**Al-Mukalla rural**	90.54	88.62	91.36	88.03	83.33	80.18	90.03	81.59
**Al-Shahr**	81.97	84.55	85.45	80.13	81.54	89.38	94.42	79.21
**Ghail Bawazeer**	89.85	89.67	81.34	81.03	80.77	84.47	100.00	91.26
**Al-Dees**	81.79	74.61	82.60	80.36	96.19	93.78	89.37	71.01
**Al-Ridh and Gosaiar**	89.52	93.33	87.55	91.04	100.00	100.00	96.46	88.39
**Ghail binYumin**	90.48	83.24	87.45	84.71	96.65	97.22	100.00	100.00
**Doan**	90.89	95.02	93.11	94.48	90.65	93.32	91.49	85.05
**Al-Dulaia**	81.82	85.91	91.37	88.16	88.01	100.00	98.92	93.81
**Hajer**	80.56	79.76	61.94	80.31	80.94	94.37	100.10	97.40
**Broome- Mayfa'a**	80.56	85.78	94.25	100.00	100.00	94.87	97.76	85.05
**Yeabeth**	83.06	86.36	87.72	86.71	79.86	80.24	90.54	100.00
**Coastal Hadhramout (Total)**	87.93	88.23	88.37	88.31	92.63	96.56	100.00	91.62
